# Research progress of kinesin family in neurological diseases

**DOI:** 10.3389/fncel.2025.1527305

**Published:** 2025-09-02

**Authors:** Shuyi Liu, Jialing Chen, Liping Shi, Yuan Deng, Zhengbo Wang

**Affiliations:** ^1^State Key Laboratory of Primate Biomedical Research, Institute of Primate Translational Medicine, Kunming University of Science and Technology, Kunming, Yunnan, China; ^2^Yunnan Key Laboratory of Primate Biomedical Research, Kunming, Yunnan, China

**Keywords:** kinesin superfamily proteins, neurodevelopment, nervous system disease, neurotumors, neurodegenerative disease, cancer

## Abstract

Kinesin superfamily proteins (KIFs) constitute a pivotal class of molecular motors that facilitate the intracellular transport of cellular “cargo.” Their principal functions encompass the participation of the transport of cellular substances along microtubules, as well as the engagement in the formation of the mitotic spindle and the segregation of chromosomes during cellular mitosis. Dysregulation of KIFs expression can precipitate anomalies in intracellular material transport, mitotic abnormalities, aberrant cell proliferation and migration, and genomic instability within cells. Moreover, members of the KIFs are implicated in the proliferation of neural progenitor cells and the migration of neurons, which are critical processes in the development of the central nervous system. To date, an extensive body of research has substantiated the close correlation between mutations or aberrant expression of KIFs and the onset of neurological disorders, including neurotumors, neurodegenerative disease, and psychiatric illnesses. This review will synthesize recent research elucidating the nexus between KIFs and neurodevelopment, as well as their association with neurological diseases.

## 1 Introduction

**FIGURE 1:**
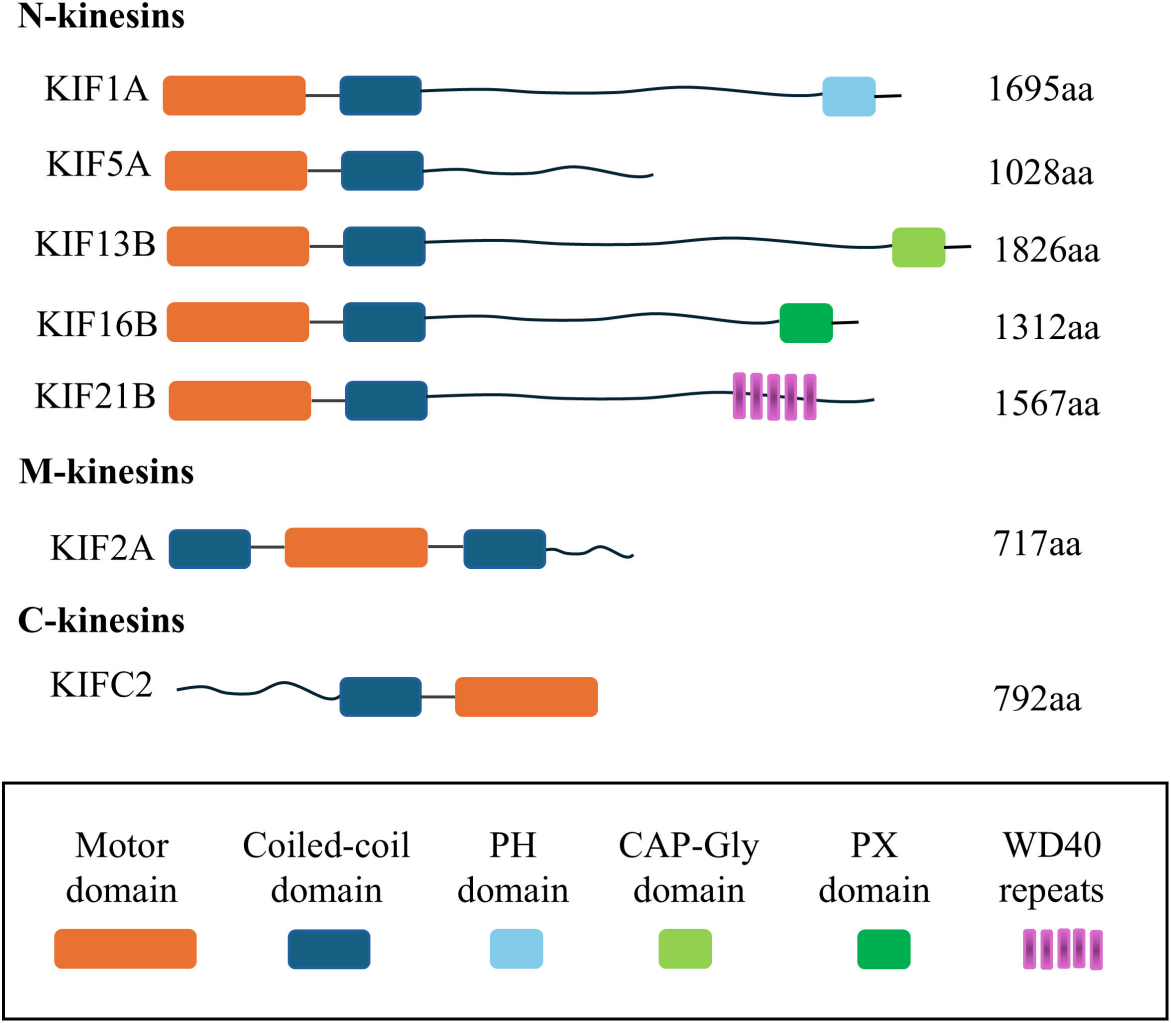
Schematic diagram of the distribution location of the motor protein domain. This figure summarizes the driver protein domains, including N-Kinesin, M-Kinesin, and C-Kinesin and the location of their motor domains, respectively: (1) N-terminal KIFs, where the motor domain is situated at the amino terminus, represent most of this subclass. These motors predominantly move toward the plus-end of microtubules to effect intracellular transport and are accordingly designated as N-KIFs. (2) C-terminal KIFs, characterized by the motor domain’s location at the carboxyl terminus, are involved in transport toward the minus-end of microtubules. This group, named C-KIFs, includes KIFC1, KIFC2, and KIFC3. (3) M-KIFs, possess a motor domain that is centrally positioned and are not engaged in the transport of cellular “cargo” along microtubules. Instead, they participate in the depolymerization of microtubules, with examples including KIF2A, KIF2B, and KIF2C. KIFs, kinesin superfamily proteins; aa, amino acid; PH, pleckstrin homology; CAP-Gly domain, cytoskeleton-associated protein-glycine-rich domain; PX, P ho x homology.

Kinesin superfamily proteins (KIFs) represent a class of molecular motor proteins that play a crucial role in intracellular transport. Initially identified in 1985, these proteins form filamentous structures interfacing with microtubules and cellular organelles, orchestrating the movement of cellular “cargo” along the microtubule network (Hirokawa et al., 1985; Vale et al., 1985). Currently, the human genome has yielded the identification of 45 distinct KIFs, which are ubiquitous across a range of tissue cells. Homologs of these proteins have also been detected in a variety of organisms, including plants, other mammals, and fungi, highlighting their evolutionary conservation and functional significance (Miki et al., 2001; Dagenbach and Endow, 2004). KIFs are categorized into 14 distinct subclasses, ranging from Kinesin1 to Kinesin14, as delineated in Table 1. It is important to note that the structural and functional attributes of these subclasses exhibit significant heterogeneity, reflecting their diverse roles in cellular transport mechanisms (Lawrence et al., 2004). The majority of KIFs are comprised of three principal structural components: a motor domain, a stalk domain, and a tail region. The motor domain interacts with microtubules and facilitates the motor’s procession along the microtubule lattice through the hydrolysis of ATP. The tail region, along with the less frequently observed stalk domain, is responsible for the recognition and binding of cellular “cargo.” Based on the positioning of the motor domain, KIFs are classified into three primary types (Hirokawa et al., 1989, 2009; Hirokawa, 1998). These distinctions are further elucidated in the schematic representation of the motor protein domain distribution depicted in Figure 1. The architectural and conformational diversity of KIFs underpins their functional repertoire (Wordeman, 2010). Primarily, KIFs are tasked with the intracellular transport of “cargo” and the preservation of cellular morphology and function by modulating the positioning and activity of functional molecules (Hirokawa et al., 2010; Hirokawa and Tanaka, 2015; Forth and Kapoor, 2017). Moreover, KIFs are implicated in cellular proliferation processes, including spindle assembly and chromosome segregation, thereby playing a pivotal role in cell cycle progression (Wordeman, 2010; Cross and McAinsh, 2014). Dysregulated expression of KIFs can precipitate a spectrum of cellular dysfunctions, including disruptions in mitotic processes, genomic instability, and aberrant cell proliferation and migration.

**TABLE 1:** Members and classification of the kinesin family.

Kinesin name	KIFs	Structure
Kinesin1	KIF5A KIF5B KIF5C	N-Kinesins KIFs
Kinesin2	KIF3A KIF3B KIF3C KIF17	N-Kinesins KIFs
Kinesin3	KIF1A KIF1B KIF1C KIF13A KIF13B KIF14 KIF16A KIF16B	N-Kinesins KIFs
Kinesin4	KIF4A KIF4B KIF7 KIF21A KIF21B KIF27	N-Kinesins KIFs
Kinesin5	KIF11	N-Kinesins KIFs
Kinesin6	KIF20A KIF20B KIF23	N- Kinesins KIFs
Kinesin7	KIF10	N-Kinesins KIFs
Kinesin8	KIF18A KIF18B KIF19A KIF19B	N-Kinesins KIFs
Kinesin9	KIF6 KIF9	N-Kinesins KIFs
Kinesin10	KIF22	N-Kinesins KIFs
Kinesin11	KIF26A KIF26B	N-Kinesins KIFs
Kinesin12	KIF12 KIF15	N-Kinesins KIFs
Kinesin13	KIF2A KIF2B KIF2C KIF24	M-kinesins KIFs (KIF2A KIF2B KIF2C); N-kinesins KIFs (KIF24)
Kinesin14	KIFC1 KIFC2 KIFC3 KIF25	C-kinesins KIFs (KIFC1 KIFC2 KIFC3); N-kinesins KIFs (KIF25)

The table contains the name of the kinesin, as well as the members and structure of the corresponding KIFs. M-KIFs, possess a motor domain that is centrally positioned and are not engaged in the transport of cellular “cargo” along microtubules. Instead, they participate in the depolymerization of microtubules, with examples including KIF2A, KIF2B, and KIF2C. KIFs: Kinesin superfamily proteins.

**FIGURE 2:**
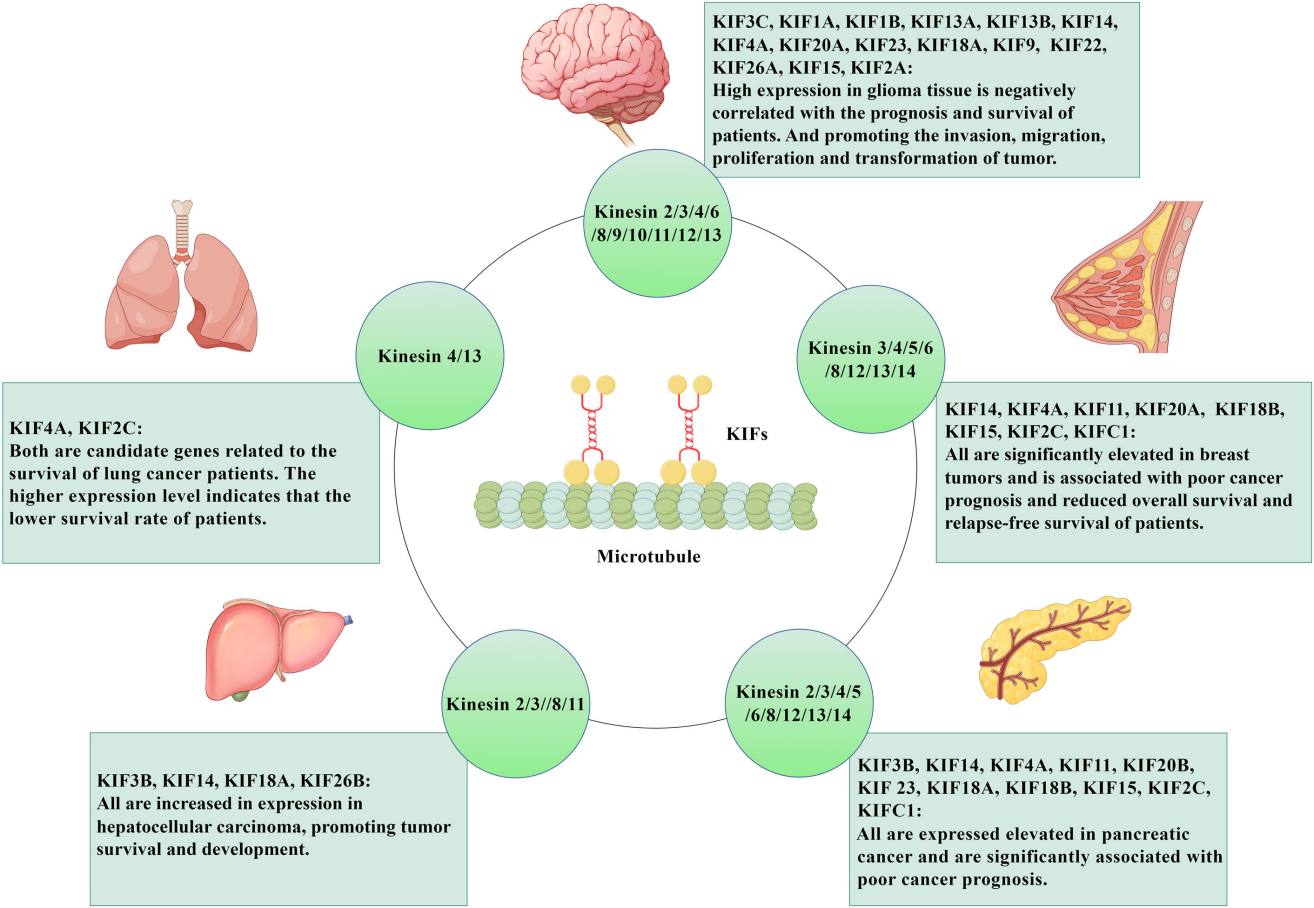
The role of KIFs in tumor diseases. KIFs are microtubule-dependent motility proteins, which are oriented along microtubule orbits and have a wide range of effects on cell functions. They also play an important role in tumor diseases. KIF3c, KIF1a, KIF1b, KIF13a, KIF13b, KIF14, KIF4a, KIF20a, KIF23, KIF18a, KIF9, KIF22, KIF26a, KIF15, KIF2a are highly expressed in glioma tissues, and are negatively correlated with patient prognosis and survival. At the same time, they also can promote the invasion, migration, proliferation and transformation of glioma. KIF4A KIF2C is predicted to be a candidate gene associated with survival in lung cancer patients, and the higher their expression levels, the lower the survival rate of patients. KIF14, KIF4a, KIF11, KIF20a, KIF18b, KIF15, KIF2c, and KIFc1 are all significantly elevated in breast tumors and are associated with poor cancer prognosis and decreased overall survival and relapse-free survival. The expression of KIF3b, KIF14, KIF18a, and KIF26b is increased in hepatocellular carcinoma tissues and promotes the survival and development of hepatocellular carcinoma. Expressions of KIF3b, KIF14, KIF4a, KIF11, KIF20b, KIF23, KIF18a, KIF18b, KIF15, KIF2c, and KIFc1 are elevated in pancreatic cancer and significantly correlated with poor prognosis of pancreatic cancer. Created with https://www.figdraw.com/#/. KIFs: kinesin superfamily proteins.

The current extensive research have shown the potential value of KIFs as an early biomarker and prognostic biomarker for a variety of cancers, and the study of drugs targeting these genes may also contribute to the treatment of corresponding cancers and improve the survival rate of patients (Gao et al., 2020a; Chen J. et al., 2021; Guo et al., 2023; Liu et al., 2023). These tumors and their corresponding KIFs are summarized in Figure 2. It mainly covers the role of these proteins in tumorigenesis and elucidation of related signaling cascades in various malignant tumors. For example, studies have shown that the expression of KIF3B (Huang et al., 2014) and KIF26B (Li et al., 2019) is significantly increased in hepatocellular carcinoma (HCC) tissues and HCC cell lines, and the knockout of KIF3B or KIF26B stimulated apoptosis of cancer cells and reduced malignant characteristics of tumors. These results suggest that they play a promoting role in the pathogenesis of HCC. KIF18A can promote HCC cell invasion and migration through Akt and MMP-7/MMP-9 related pathways and may promote HCC cell proliferation by promoting cyclin B1 expression (Luo et al., 2018). In addition, silencing KIF14 can save advanced resistance to HCC chemotherapy drugs (Zhu et al., 2020). In conclusion, KIFs plays an important role in HCC, and studying the influence of expression status of different KIFs subtypes on tumors can help the development of anti-tumor drugs for HCC. In addition to HCC, KIFs also plays an important role in pancreatic cancer and pancreatic ductal adenocarcinoma. KIF3B is highly expressed in human pancreatic cancer tissues, which is significantly associated with poor prognosis such as pTNM staging, lymph node metastasis and vascular invasion (Liu et al., 2019). Another study shows that KIFC1/2C/4A/11/14/15/18A/18B/20B/23 are up-regulated in patients with pancreatic cancer and is associated with adverse clinical outcomes (Yang et al., 2021). In addition, abnormal expression of KIFs has been found in breast and lung cancer. Fourteen microtubule-related proteins genes (KIF4A, ASPM, KIF20A, KIF14, TPX2, KIF18B, KIFC1, AURKB, KIF2C, GTSE1, KIF15, KIF11, RACGAP1, STMN1) are significantly upregulated in breast tumors. Six of the genes (KIF4A, ASPM, KIF20A, KIF14, TPX2, KIF18B) are overexpressed by more than 10-fold. High expression of these genes is associated with poor clinical outcomes in patients, who show reduced overall survival and relapse-free survival (Rodrigues-Ferreira et al., 2023). In lung cancer, Huang et al. (2023) identify seven candidate genes associated with survival of lung cancer patients with bioinformatics analysis, namely TOP2A, TK1, KIF4A, ANLN, KIF2C, ASF1B, and CCNB1. The data shows that lung cancer patients with relatively high expression levels of the seven candidate genes have lower survival rates than patients with relatively low expression levels. And down-regulated KIF2C expression will inhibit the proliferation, migration and invasion of non-small cell lung cancer cells, and promoted apoptosis (Huang et al., 2023).

In addition to extensive research in cancer, based on the diversity of KIFs functions, current research is also beginning to focus on the relationship between KIFs and the central nervous system. At present, much research has substantiated the correlation between mutations and aberrant expression patterns of KIFs with impaired neural development in the brain. These anomalies have been implicated in the pathogenesis of neurodegenerative conditions and psychiatric disorders. This review aims to delineate the role of KIFs within the brain, and their nexus with the evolution of neurological diseases.

## 2 Function of KIFs in neurodevelopment

The ontogeny of the brain encompasses critical developmental milestones, including the proliferation and differentiation of neural progenitor cells, the migration and maturation of neurons, and the establishment of synaptic connections. Each of these stages is pivotal to the orchestration of brain development, with the intricate interplay of multiple genes and regulatory factors. Deviations at any juncture can precipitate cerebral dysfunction and a spectrum of neurological disorders. The KIFs are instrumental in cellular mitosis, proliferation, and migration, and accumulating evidence suggests that certain members of the KIFs are integral to the regulatory mechanisms governing the development of the brain and nervous system.

### 2.1 KIFs are involved in proliferation and differentiation of neuron

Several members of the KIFs are implicated in the proliferative processes of neural progenitor cells. Notably, KIF20B, a constituent of the Kinesin-6 subfamily, exhibits robust expression in embryonic neural progenitors. Disruption of its functional capacity has been shown to diminish the proliferation of both neural progenitor cells (NPCs) and neuronal lineages within the cerebral cortex. Consequently, this reduction culminates in a cortical layer that is proportionally thinner compared to the norm, highlighting the indispensable role of KIF20B in the context of neural development (Janisch et al., 2013). KIF20A, another member of the Kinesin-6 family, has been identified as playing a crucial role in maintaining the proliferative state of NPCs during the development of the cerebral cortex. Knockout studies of KIF20A have demonstrated that NPCs in the cerebral cortex transition from a proliferative and dividing state to a differentiated state, which is accompanied by the apoptosis of progenitor cells and neurons. This shift ultimately leads to cortical thinning and ventriculus cerebri enlargement. Notably, partial loss of KIF20A function results in the exit of neurons from the cell cycle and the premature differentiation of neurons; however, it does not induce significant defects in cell division or apoptosis (Geng et al., 2018; Qiu et al., 2020). Other members of KIFs, including KIF15 (Feng et al., 2016) and KIF3A (Tong et al., 2014; Feng et al., 2016; Cullen et al., 2021), have been implicated in the proliferation and differentiation of neural progenitor cells. Furthermore, according to the study published by Naher et al. (2025), KIF23 was a key molecule regulating neural stem cell development, and its loss of function can directly lead to the pathological mechanism of microcephaly. The study of Naher et al. (2025) not only reveals the key role of KIF23 in maintaining the neural stem cell pool during embryonic brain development but also provides a molecular basis for the genetic diagnosis and treatment of microcephaly through the elucidation of the pathogenic mutation mechanism. This suggests that future studies need to further explore the function of KIF23 in primate models and the window of clinical intervention to promote the transformation from mechanism discovery to treatment (Naher et al., 2025). These findings underscore the significant role of KIFs in cerebral development, particularly in the context of the proliferation and differentiation of neural progenitor cells within the cerebral cortex. The functional involvement of these kinesin family members during neurogenesis highlights their potential as key regulatory factors in the construction and refinement of the neural architecture.

### 2.2 KIFs and neuron migration

Neuronal migration denotes the orchestrated relocation of immature neurons through time and space, ensuring their accurate arrival at their predetermined functional destinations within the neural architecture. This process is meticulously regulated and is fundamental to the proper formation and functionality of neural circuits. KIFs are also instrumental in the process of neuronal migration, highlighting their essential roles in the orchestration of neuronal positioning and the subsequent refinement of neural circuits.

KIF2A is an ATP-dependent microtubule depolymerizing factor that plays a critical role in regulating microtubule dynamics. Specifically, the targeted ablation of KIF2A in inhibitory forebrain neurons leads to aberrations in the tangential migration of interneurons within the cortex, subsequently affecting their proper cortical localization (Ruiz-Reig et al., 2022a). Additionally, it has been observed that the downregulation of KIF20B levels impedes the transition of multipolar cells from a multipolar to a bipolar state in the ventricular zone, thereby inhibiting neuronal migration. These findings underscore the significance of KIF20B in the dynamic processes underlying neuronal polarization and migration (Sapir et al., 2013). Furthermore, additional research into the developing rodent brain has revealed that KIF15 is markedly enriched in migrating neurons relative to their non-migrating counterparts (Buster et al., 2003). Subsequent studies have demonstrated that KIF15 directly interacts with myosin-IIB through its tail domain, thereby modulating the proliferation of neuronal precursor cells and the migratory behavior of immature neurons. This protein is not only implicated in neuronal migration but also plays a role in the migration of astrocytes (Feng et al., 2016). KIFC1, a member of the Kinesin14 family, is capable of propelling microtubule sliding, which facilitates the directional rotation of the nucleus during neuronal migration, thereby ensuring that these cells remain on their intended trajectory. The absence of KIFC1 leads to deviations in the migratory path of neurons, highlighting the essential role of KIFC1 in maintaining the fidelity of neuronal migration trajectories (Muralidharan et al., 2022). Collectively, these findings underscore the significant impact of KIFs on the migratory processes of neurons.

### 2.3 KIFs influence the morphology and function of neurons

Kinesin superfamily proteins are principal molecular motors protein that play a crucial role in intracellular transport. Specific members of the KIFs are tasked with the conveyance of assorted “cargos” along microtubules to the axonal and dendritic compartments of neurons. This vesicular transport is integral to the modulation of neuronal status and cerebral function, encompassing the regulation of “cargo” trafficking, as well as the growth and functional sustenance of axons and dendrites.

KIF1A is predominantly engaged in the axonal transport of synaptic vesicle precursors (SVPs) along microtubules, exerting a pivotal role in the viability and functional preservation of mature neurons (Okada et al., 1995). Yonekawa et al. (1998) found that, in neurons with KIF1A mutations, the transport of SVPs is markedly diminished, culminating in an inability to adequately receive afferent stimuli, inclusive of neuronal contact and neurotransmission. This deficiency ultimately precipitates neuronal demise (Yonekawa et al., 1998). However, another study found that the KIF1A mutation associated with hereditary spastic paraplegia would lead to excessive activation of KIF1A movement, thereby causing abnormal accumulation of SVPs at axon tips, and increases the anterograde axonal transport of SVPs. This change can disrupt the homeostasis of human motor neurons and is one of the causes of motor neuron diseases (Chiba et al., 2019). Niwa et al. (2008) first identified KIF1Bβ as working together with KIF1A to mediate the axonal antegrade transport of SVPs. Subsequently, Xu et al. (2018) further revealed that KIF1Bβ undertakes a variety of cargo transport tasks in neurons, beyond the category of synaptic vesicles, including mitochondrial transport and the transport of neurotrophin-receptor TrkA. In summary, the synergistic action of KIF1A and KIF1Bβ ensures efficient synaptic vesicle trafficking, and the independent function of KIF1Bβ in mitochondrial and TrkA receptor trafficking further expand the regulatory dimension of kinesins in neuronal survival and disease. Within the same KIFs, the ablation of KIF1B elicits aberrations in synaptic transport and is associated with impaired axonal elongation (Xu et al., 2018). The primary role of KIF17 within the Kinesin2 family is to facilitate the intracellular trafficking of the *N*-methyl-D-aspartate receptor NR2B subunit, the metabotropic glutamate receptor 5, and the potassium channel Kv4.2 from the cell soma to the dendrites. This transport process is pivotal for cognitive functions such as learning and memory. Subsequent research has corroborated that the absence or dysfunction of KIF17 leads to deficits in the learning capabilities and spatial memory retention of rodents (Wong et al., 2002; Wong-Riley and Besharse, 2012; Yin et al., 2012).

Dysfunctional KIFs members can significantly impact on neuronal morphology. Neurons deficient in KIF2A exhibit aberrant electrophysiological properties and atypical growth patterns of dendritic collaterals (Homma et al., 2003, 2018; Akkaya et al., 2021; Ruiz-Reig et al., 2022b). The downregulation of KIF3B in neurons results in an excessive proliferation of dendritic spines and branches, suggesting that KIF3B plays an inhibitory and regulatory role in the structural plasticity of neurons (Joseph et al., 2020). Furthermore, KIF3B knockdown has been associated with impaired contextual fear memory in mice (Alsabban et al., 2020; Joseph et al., 2021; Yoshihara et al., 2021). In contrast, the knockdown of KIF3A, KIF5 (comprising KIF5A, KIF5B, and KIF5C), and KIF11 has been shown to have the opposite effect to KIF3B, leading to a reduction in dendritic branching and spine density (Liu et al., 2010; Zhao et al., 2020; Lucero et al., 2022). KIF5C is a key regulator of excitatory synaptic transmission, structural plasticity, and local translation. Experiments have shown that the loss of KIF5C function in dorsal CA1 neurons of horses is related to the attenuation of spatial and situational fear memory, because this process reduces dendritic branch formation and spinal density, which affects the learning and memory ability of the body (Swarnkar et al., 2021). Recent studies have revealed that the deletion of KIF2C can significantly alter the density of dendritic spines within the hippocampus of mice, resulting in impaired excitatory synaptic transmission and alterations in cognitive behavior (Zheng et al., 2022). Our research group has conducted corroborative studies on the role of KIF2C, which have substantiated these findings. Building upon this foundation, we have discovered that the absence of KIF2C also leads to aberrant dendritic branching and spine formation in the cerebral cortex of mice, consequently impacting their cognitive function. Furthermore, we intend to delve deeper into the mechanistic underpinnings of these phenomena through a comprehensive investigation. Collectively, these findings underscore the indispensable role of KIFs in the morphological and functional regulation of neuronal integrity.

## 3 KIFs and neurological disorders

As summarized in the previous section, KIFs play important functions in various aspects of brain development, including neuronal proliferation and differentiation, migration, and functional regulation. Alterations in KIFs genes, such as mutations or downregulations, can make these processes abnormal, resulting in brain dysfunction. There is abundant research evidence showing that KIFs are closely related to the occurrence of various diseases in the brain, and details can be found in Table 2.

**TABLE 2:** Kinesin superfamily proteins (KIFs) and neurological diseases.

Disease	Participating KIFs members	Relevant research points	References
Glioma	KIF1A, KIF1B, KIF2A, KIF3C, KIF4A, KIF9, KIF13A, KIF13B, KIF14, KIF15, KIF18A, KIF20A, KIF22, KIF23, KIF26A	KIF3C may inhibit tumor growth by activating the PI3K/AKT/mTOR signaling pathway, thereby prolonging the survival time of patients.	Gao et al., 2020c; Yan et al., 2024
Meningioma	KIFC1, KIF4A, KIF11, KIF14, KIF20A	KIF11 inhibitors inhibit the growth of meningiomas in xenografted mice by more than 80% and have few blood side effects.	Jungwirth et al., 2021
Stroke	KIF2	KIF2C may mediate neuroprotection from cerebral ischemia/reperfusion injury by inhibiting activation of the NF-κB pathway	Wang et al., 2020
Alzheimer’s disease	KIF2C, KIF4A, KIF5, KIF11, KIF12, KIF18B, KIF21B	Aβ inhibits mitotic motor proteins such as KIF4, Eg5 and MCAK, disrupting the function and plasticity of neurons.	Borysov et al., 2011
Amyotrophic lateral sclerosis	KIF5A	The ALS fruit fly model of KIF5A mutant basically covers the characteristics of the disease, and KIF5A mutant exists in cytoplasmic inclusion bodies, and the pathogenicity comes from the toxic acquisition of function.	Nicolas et al., 2018; Soustelle et al., 2023
Multiple sclerosis	KIF1B, KIF5A, KIF21B	KIF5A levels in CSF were positively correlated with MS disease severity score and could be used as a predictive biomarker for MS.	Hares et al., 2021
Hereditary spastic paraplegia	KIF1A, KIF13B	KIF1A variation is a common cause of autosomal dominant HSP, and KIF1A loss variation may be the pathogenic mechanism of HSP.	Pennings et al., 2020
Charcot-marie-tooth disease	KIF5A	The absence of KIF5A causes CMT	Pyromali et al., 2021
Autism spectrum disorder	KIF1A, KIF22	The KIF22 mutant in the Drosophila model of ASD causes ectopic innervation of the axon branch of muscle 13 to form a type III button.	Park et al., 2016; Huang et al., 2021
Microcephaly	KIF14, KIF11, KIF2A	The KIF11 mutation was found in 75% of patients with microcephaly in clinical samples; KIF14 mutations have also been reported in patients.	Balikova et al., 2016; Cavallin et al., 2017; Malvezzi et al., 2018
Intellectual disability	KIF16A, KIF4A, KIF5C	KIF16B, KIF4A, and KIF5C mutations were found in clinical samples, and patients showed varying degrees of intellectual disability.	Okamoto et al., 2017; Alsahli et al., 2018
Schizophrenia	KIF2, KIF3, KIF7, KIF13A, KIF26B	Up-regulation of KIF3 was found in patient samples, and KIF2 and KIF13A were also considered genetic susceptibility genes for schizophrenia.	Alsabban et al., 2020; Zhang and Chen, 2020; Sargazi et al., 2021; Yoshihara et al., 2021

The table summarizes the association of KIFs with neurological diseases, including the disease, the KIFs members involved with the disease, and the relevant research points. Detailed information is attached to the references.

### 3.1 Brain tumors

Abnormal expression of KIFs can lead to cell mitosis disorder and cell genomic instability. Genome instability, in particular, is a key factor in transformation of cells into cancer and tumor. Many clinical data have confirmed the involvement of KIFs in the occurrence and development of various malignant tumors (Chen et al., 2017; Lucanus and Yip, 2018; Ghnim et al., 2024; Yang et al., 2021). Schlisio et al. (2008) first established that KIF1Bβ is a key tumor suppressor gene in neuroblastoma, and the expression of KIF1B is significantly downregulated or even absent in a variety of neural crest derived tumors such as neuroblastoma, pheochromocytoma, and glioma. KIFs members have been found to play significant role in the disease progression of brain tumors.

Glioma is one of the most common primary malignant tumors in adults, mainly occurring in brain and glial tissues, and has a very high mortality due to its localization and powerful invasive ability (Ostrom et al., 2014). The median survival of glioma patients is only 14–16 months (Louis et al., 2016). It is classified into grades 1–4 according to the degree of malignancy. KIFs members (KIF1A, KIF1B, KIF2A, KIF3C, KIF4A, KIF9, KIF13A, KIF13B, KIF14, KIF15, KIF18A, KIF20A, KIF22, KIF23, KIF15, KIF18A, KIF20A, KIF22, KIF23, KIF26A) are highly expressed in glioma tissues (Wang et al., 2013; Chen et al., 2016; Sun et al., 2016; Zhang et al., 2016, 2022; Saito et al., 2017; Cho et al., 2019; Gao et al., 2020b; Iegiani et al., 2021; Ren et al., 2022; Tao et al., 2022; Pan et al., 2023), and are more expressed in glioma tissues with high malignant degree (Zhang et al., 2016). Moreover, the expression level of KIF is negatively correlated with the prognosis and survival of patients (Chen et al., 2016). Subsequent studies have found that downregulate the expression levels of these KIF members can inhibit the proliferation, invasion and migration of glioma cell lines (Saito et al., 2017; Gao et al., 2020b; Zhang et al., 2022). For example, KIF3C may promote the proliferation, migration and invasion of glioma cells by activating the PI3K/AKT/mTOR pathway *in vitro*, inhibit the apoptosis of cancer cells, and prolong survival time. Inhibition of KIF4A can inhibit the progression of glioma, and addition of a small molecule inhibitor of KIF4A induced apoptosis in glioma cells, showing anti-glioma effects. These results suggest that these KIFs members may become new predictors and therapeutic targets for gliomas.

Meningiomas (MGMs) are also one of the most common types of central nervous system tumor, accounting for one-third of all brain tumor (Mawrin and Perry, 2010). MGMs are histologically classified according to the WHO grading scheme: nearly 80% of MGMs are benign, corresponding to WHO°I, 10%–15% are atypical (WHO°II), and only 2%–5% are anagenic MGMs WHO°III (Louis et al., 2007). Among them, the survival rate of benign MGM patients is 92%, the survival rate of atypical MGM drops to 78%, and the survival rate of WHO° Grade III MGM patients is only 47% (Durand et al., 2009; Vranic et al., 2010; Gousias et al., 2016). Durand et al. (2009) analyzed a microarray dataset of benign, atypical, and anaplastic MGMs and obtained differential expression of five driver proteins (KIFC1, KIF4A, KIF11, KIF14, and KIF20A) that are significantly upregulated in WHO°I to°III MGMs. Later studies find that knocking down KIF11 in anaplastic MGM cell line NCH93 inhibited 71% of tumor cell proliferation (Jungwirth et al., 2019). Subsequent studies have shown that KIF11 inhibitors filanesib and ispinesib can induce G2/M stagnation in all MGM cell lines and significantly inhibit the growth rate of MGM in mice *in vivo* up to 83% (Jungwirth et al., 2021), indicating that inhibiting the function of KIF11 may be one of the ways to treat MGMs.

### 3.2 Stroke

Stroke is an acute cerebrovascular disease, which is a group of diseases caused by brain tissue damage due to the sudden rupture of blood vessels in the brain or the blockage of blood vessels that prevents blood from flowing to the brain, including ischemic and hemorrhagic stroke. The onset age of stroke is more over 40 years old, and has long been a major cause of death in China (Wang et al., 2024). O’Connell et al. (2017) obtain publicly available whole blood microarray data generated 3, 5, and 24 h after symptom onset in 23 ischemic stroke patients, and 23 cardiovascular disease controls using the National Center for Biotechnology Informatics Gene Expression Synthesis. There are 10 candidate genes with expression levels that distinguish stroke patients from controls, including KIF1B (O’Connell et al., 2017). The other study has found that KIF2 is abnormally expressed in the brain tissue of middle cerebral artery occlusion rat model in a time-dependent manner, and inhibition of KIF2 significantly increases the level of malondialdehyde in the brain tissue of middle cerebral artery occlusion model and decreases the activities of superoxide dismutase and glutathione peroxidase. Increase the severity of the disease. It is also found that KIF2-mediated nuclear factor-kappa B (NF-κB) pathway is involved in neuroprotection after cerebral ischemia/reperfusion injury, and the addition of NF-κB pathway inhibitors can reduce the apoptosis of KIF2-silenced hypoxic cells (Wang et al., 2020). This may provide a new diagnostic indicator and therapeutic target for strokes.

### 3.3 KIFs and neurodegenerative diseases

#### 3.3.1 Alzheimer’s disease

Alzheimer’s disease (AD) is one of the most common neurodegenerative diseases. Its main pathological features are excessive deposition of β-amyloid peptide (Aβ) and formation of plaques, resulting in neurotoxicity and further causing degenerative brain dysfunction (Meyer-Luehmann et al., 2008; Burns and Iliffe, 2009). Morihara et al. (2014) revealed that alternative splice variants of kinesin-light chain 1 (KLC1), such as KLC1-E, are key factors for Aβ pathological deposition, which further confirmed the association between KIFs and AD. Aβ is produced by the enzymatic cleavage of amyloid precursor protein (APP), and KIF5 is involved in the axonal transport of APP in cells, and there is a negative correlation between APP level and KIF5A level (Hares et al., 2017; Chen X. et al., 2021). This suggests that reduced KIF5 levels may cause abnormal axonal transport of APP, leading to Aβ accumulation at specific locations. In AD patients and their animal models, excessive deposition of Aβ directly disrupts the assembly and maintenance of the mitotic spindle, which then leads to chromosomal missegregation and aneuploidy. Approximately 30% of aneuploid/superploid neurons that occur in AD are particularly prone to degeneration and may account for 90% of neuronal loss in advanced AD (Arendt et al., 2010). These defects are most likely caused by Aβ inhibition of mitotic driver proteins, including KIF11, KIF4A, and KIF2C (Borysov et al., 2011). Among them, the inhibition of KIF11 function by Aβ can inhibit the transport of nerve growth factor/neurotrophin receptor (NGF/NTR, p75) and N-methyl-D-aspartic acid receptor on the cell surface (Ari et al., 2014), and these neurotransmitters and neurotrophin receptors are key to the regulation of learning and memory ability. Further studies found that overexpression of KIF11 can rescue spatial learning deficits in AD mouse models (Lucero et al., 2022). Therefore, the mechanism of Aβ-induced neuronal dysfunction may underlie the learning and memory deficits in AD, and prevention of Aβ-induced inhibition of KIFs members may become a new treatment option for AD.

Several studies that analyzed genetic and metabolic samples of clinical AD patients have found that multiple KIFs members may be closely associated with the development of AD. The studies have compared plasma peptides and proteins in dementia patients with normal controls and found that KIF12 protein peptide is observed more frequently in dementia patients (Florentinus-Mefailoski et al., 2021). Through microarray analysis, circ-KIF18B is positively correlated with Aβ42 in AD patients (Li et al., 2020). Other researchers evaluated the expression level of driver protein in AD patients after death, and found that compared with the control group, the expression of KIF21B in AD patients under 62 years old increased by 5 times, and in AD patients between 62 and 72 years old increased by three times (Kreft et al., 2014; Le Guen et al., 2021). These results indicate that KIF12, KIF18B, and KIF21B have potential as biomarkers for AD, which may be helpful for clinical diagnosis of AD patients.

#### 3.3.2 Amyotrophic lateral sclerosis

Amyotrophic lateral sclerosis (ALS) is a fatal adult-onset neurological disease characterized by degeneration of the upper and lower motor neurons of the motor cortex, brain stem, and spinal cord, leading to progressive atrophy and paralysis of skeletal muscles. About 10% of ALS are familial, and about 20% of these familial ALS cases are associated with mutations in the copper/zinc SOD1 gene. The remaining 90% are sporadic. Brenner et al. (2018) used whole exome sequencing to perform genetic analysis on 426 familial ALS patients and 6,137 control subjects in Europe, and detected mutations in the C-terminal of KIF5A (p.Arg27Gly, P.Ro986leu) that could cause typical ALS phenotypes. Zhang et al. (2019) also analyzed the ALS cohort in China and found A KIF5A splicing site mutation c.2993-1G > A in one sporadic ALS patient. Other researchers have found that patients with the KIF5A lost-of-function mutation have relatively longer survival compared to other typical ALS cases (Nicolas et al., 2018). Current research data have proved that KIF5A is a related gene to ALS, and subsequent studies on the functional regulation and pathogenic mechanism of KIF5A in the brain may help to develop new sites and new ways of ALS treatment (Baron et al., 2022; Nakano et al., 2022; Pant et al., 2022).

#### 3.3.3 Multiple sclerosis

Multiple sclerosis (MS) is a neurodegenerative disease of the central nervous system, one of the most common diseases in young people, affecting more than 2.5 million people worldwide. MS is characterized by central nervous system inflammation, demyelination, axonal degeneration, and neuronal damage. In 2008, through the mutation analysis of 2,679 MS cases and 3,125 controls, it is found that the variation of rs10492972[C] in KIF1B gene is highly correlated with MS (Aulchenko et al., 2008). Subsequently, Hares et al. (2014) analyzed 27 MS cases and 13 control cases unrelated to neurological diseases in 2014, and find that mRNA expression of KIF5A, KIF21B, and KIF1B and KIF5A protein expression in the gray matter of MS patients are significantly reduced compared with controls. Another study finds that MS patients with high expression of KIF21B have a 2-fold accelerated rate of disease progression (median time is 16 years for the low KIF21B group and 7.5 years for the high KIF21B group). In subsequent studies, KIF21B is found to be correlated with the degree of gray matter demyelination in MS patients (Kreft et al., 2014). These studies on the correlation between KIFs and MS demonstrate the involvement of KIFs in the disease process and suggest that KIFs are expected to become a clinical diagnostic indicator and therapeutic target.

#### 3.3.4 Hereditary spastic paraplegia

Hereditary spastic paraplegia (HSP) is a rare heterogeneous inherited neurodegenerative disease characterized by progressive spastic weakness (stiffness) of the lower limbs due to axonal degeneration of the corticospinal tracts, and can be classified as either simple HSP or HSP with neurological abnormalities (complex HSP) (Lo Giudice et al., 2014). In complex cases, HSP may be accompanied by other neurological abnormalities such as mental retardation, peripheral neuropathy, ataxia, distal muscular atrophy, and optic neuropathy. In a study of the correlation between HSP and KIFs, mutations at the p.R204, p.N256, and p.R280 loci of the KIF5A gene are found to be associated with typical HSP symptoms (Carosi et al., 2015), and mutations at different loci are accompanied by a number of other symptoms, including sensorimotor neuropathy or cerebellar ataxia (Tessa et al., 2008; Liu et al., 2014; Cuchanski and Baldwin, 2018; Hong et al., 2023). Other researchers have analyzed the genomes of two consanguineous families with complex HSP (concomitant mental retardation, epilepsy) and found that there is a large deletion on 8p12-p11.21, and that the KIF13B gene, which is located within this interval, may be a functional candidate gene for this form of HSP (Al-Yahyaee et al., 2006).

In addition, KIF1A mutations have recently been detected in patients with HSP. A novel KIF1A mutation (c.37C > T) is identified in a male with autism, attention deficit hyperactivity disorder, epilepsy, and mild intellectual disability, sensory impairment, and spastic paraplegia (Kurihara et al., 2020). In a genetic analysis of Taiwanese patients with HSP, three different KIF1A mutations are identified in three patients with autosomal dominant HSP. Among them, patients carrying KIF1A (p.G321D) show pure HSP, while patients carrying KIF1A (p.E19K, p.R316Q) show compound HSP with axonal sensory-motor polyneuropathy. One of the patients carrying KIF1Ap. R316Q also exhibits thoracic girdle atrophy, thinning of the corpus callosum, and high white matter signal (Hsu et al., 2022). KIF1A variants are found to be a common cause of autosomal dominant HSP in research cohorts (6%–7%) (Pennings et al., 2020). These studies suggest that the loss-of-function variant of KIF1A may be a mechanism in the HSP pathogenesis.

#### 3.3.5 Charcot-marie-tooth disease

Charcot-maryoatrophy (CMT), also known as hereditary motor and sensory neuropathy (HMSN), is the most common hereditary peripheral neuropathy. Characterized by progressive distal muscle weakness, sensory loss, and loss of reflexes in the upper and lower extremities, CMT is usually classified as demyelination (CMT1), and motor nerve conduction velocities (MNCV) reduced to less than 38 m/s. The MNCV of the axonal deficient (CMT2) is 38 m/s or higher (Fridman et al., 2015). Nam et al. (2018) identify three pathogenic KIF5A mutations (p.Arg204Trp, p.Arg280His, and p.Leu558Pro) in Korean CMT2 patients by whole exome sequencing, but not all mutations are observed in healthy controls. Where the p.Arg204Trp mutation is identified from CMT2 patients with other complex phenotypes such as HSP, ataxia, fatigability and pyramidal signs (Nam et al., 2018). Other researchers have found that mutations in KIF1B also cause the peripheral neuropathy known as CMT2A (Mok et al., 2002), and subsequent studies have found defective SVPs in kif1b ± mice and suffered from progressive muscle weakness similar to CMT2A (Zhao et al., 2001). These studies suggest that synaptic transport abnormalities caused by mutations or deletions of KIFs members may be one of the pathogenic mechanisms of CMT. Subsequent in-depth studies can be conducted to reveal the pathogenic mechanism behind it, which can contribute to the discovery of new targets for the clinical treatment of CMT.

### 3.4 KIFs and neurodevelopmental disorders

#### 3.4.1 Microcephaly

Microcephaly (MCPH) is a neurodevelopmental disorder characterized by a pathological condition where the skull of a child is significantly smaller than the average size of a normal person of the same age. If the head circumference of the newborn is lower than the average head circumference of normal newborns (34 cm) by three standard deviations (about 5%), it can be diagnosed as microcephaly (De Bie and Boucoiran, 2019). Among them, autosomal recessive primary microcephaly is a MCPH characterized by reduced cerebral cortex and intellectual disability. Study shows that homozygous mutations of c.263T > A, c.2480_2482delTTG, and c.4071G > A in KIF14 have been found in three MCPH families, and KIF14 c.2545C > G and c.3662G > T complex missense mutations have been found in a patient with severe MCPH (Moawia et al., 2017). Another study shows that four mutations of KIF14 (P.SN83ILEFS *3 and P.SN1478FS: loss of function, P.er841phe and P.LY459arg: missense substitution) are present in MCPH patients with intellectual impairment (Makrythanasis et al., 2018). And KIF14 gene knockout mice also show primary MCPH (Moawia et al., 2017; Makrythanasis et al., 2018). The aforementioned results indicate that the loss of KIF14 function may represent one of the pathogenic factors contributing to MCPH. Another type of autosomal dominant primary microcephaly is characterized by primary microcephaly accompanied by lymphedema, chorioretinal dysplasia, and intellectual disability. And KIF11 mutations are found to occur in approximately 75% of patients diagnosed with this type of MCPH in clinical samples (Balikova et al., 2016). A subsequent study finds a 209kb microdeletion at 10q23.33 in a father and two children, all of whom are patients with MCPH, which contains the entire KIF11 gene (Malvezzi et al., 2018). This suggests that the impaired function of KIF11 caused by mutation and deletion of KIF11 is causally related to MCPH. And several other KIFs member mutation sites have also been detected in patients with MCPH, such as KIF2A (p.Ser317Asn and p.His321Pro) (Cavallin et al., 2017), KIF26B (p.Gly546Ser) (Wojcik et al., 2018).

#### 3.4.2 Intellectual disabilities

Intellectual disabilities (ID) is a neurodevelopmental disorder characterized by severe deficits in intelligence and adaptive behavior. About 1%–3% of the global population is affected that result in huge social and economic impacts. Okamoto et al. (2017) find new mutations in the KIF16A gene in patients with severe ID, epilepsy, acquired microcephaly, and blindness with complex phenotypes, and depletion of KIF16A or overexpression of the KIF16A mutant with truncated C-ends will lead to spindle assembly defects in human cultured cells. It is inferred that mutations in KIF16A result in abnormal spindle morphology, which subsequently leads to irregular cell division and proliferation. This cascade of events ultimately gives rise to a novel genetic syndrome characterized by ID (Okamoto et al., 2017). Alsahli et al. (2018) find a novel mutation in the KIF16B gene (c.3611T > G) in two brothers with ID. A subsequent study finds destructive mutations in KIF4A in a family of X-linked ids (c.1489-8_1490delins10, p.? - exon hopping), patients display mild to moderate ID and epilepsy. And female patients with KIF5C missense mutations (c.11465A > C, p.Glu237Lys) present with severe ID, epilepsy, microcephaly, and cortical malformations (Willemsen et al., 2014). These findings suggest a strong link between members of the KIFs and ID.

#### 3.4.3 Autism spectrum disorder

Autism spectrum disorder (ASD) is a class of neurodevelopmental disorders occurring in early infants and young children, the core characteristics of which are persistent social interaction and communication disorders, narrow interests and repetitive stereotyped behaviors (Battle, 2013; First, 2013). Tomaselli et al. (2017) conduct gene exome sequencing on a patient with ASD, spastic paraplegic and axonal neuropathy complex phenotypes, and find a dominant missense variant (c.38G > A) in the ATP binding site of KIF1A gene. Other studies have found that 16p11.2 microduplication or microdeletion is involved in about 1% of ASD cases, and KIF22 gene is located in the 16p11.2 interval, which can provide reference for the screening of ASD genetic susceptibility genes (Morson et al., 2021). Anazawa et al. (2022), Chai et al. (2024) analyzed an autism mutation, KIF1A(R11Q), and showed that synaptic vesicle transport is affected by ASD mutation. The mutation causes HSP as well. Rett syndrome (RTT) is a form of ASD that predominantly affects females, with an incidence rate of approximately 1 in 10,000 women. 95% of patients with typical RTT and 73.2% of patients with RTT variants are found to have the MECP2 pathogenic variant. A neonatal pathogenic variant of KIF1A(c.275_276ins AA, p.Cys92*) is found in a girl with all core features of typical RTT, which is the first time that a pathogenic variant of KIF1A has been associated with RTT syndrome (Wang et al., 2019).

### 3.5 KIFs and psychiatric disorders

Schizophrenia (SCZ) is a major psychiatric disorder affecting many aspects of behavior and cognition, with a global lifetime prevalence of 1% (Andreasen, 1995). According to the neurodevelopmental hypothesis of the pathogenesis of SCZ, neuronal dysplasia occurring at various stages of neurodevelopment will predispose individuals to SCZ (Birnbaum and Weinberger, 2017). Studies have shown that cerebral anatomical will change in patients with SCZ, such as reduced cortical volume, ventricular enlargement, and reduced cortical gray matter (Kochunov and Hong, 2014). In addition, clinical autopsy finds that the expression level of KIF3 is down-regulated in the prefrontal cortex of SCZ patients after death, and Kif3b^±^ mice show obvious schizophrenia traits such as social disturbance and postsynaptic defects in follow-up studies (Alsabban et al., 2020; Yoshihara et al., 2021). Other researchers conduct differential gene screening on a large number of SCZ patients from Iran and Shandong, China, and find that the KIF26B mutant may be a novel genetic biomarker of SCZ susceptibility in these two places (Zhang and Chen, 2020; Sargazi et al., 2021). Analysis of some SCZ family samples also find that KIF2 gene in the 5q12.1 region (Li et al., 2006) and KIF13A (Jamain et al., 2001) in the 6p23 region may also be candidates for genetic susceptibility genes for SCZ.

Kinesin superfamily proteins may also be related to suicidal tendencies. Niculescu et al. (2015) analyzed the blood samples of patients with suicidal tendencies, such as bipolar disorder, major depression, schizoaffective disorder and SCZ, and find that KIF2C is significantly reduced in the blood samples of patients with strong suicidal tendencies. In addition, Cabrera-Mendoza et al. (2020) evaluate the differences in DNA methylation between individuals with high and low suicidal tendencies, then find that KIF7 is differentially expressed between the two groups. These two studies indicate that the differential expression of KIFs has certain reference significance for the prediction and diagnosis of clinical suicidal tendency. Which can increase the observation and protection of suspected patients, reduce the suicide rate, and conduct in-depth research on whether it is a pathogenic factor and develop symptomatic treatment.

## 4 KIFs and rare diseases

KIF7 mutations have been found to cause Jobert syndrome, hydrolethalus syndrome, and acrocallosal syndrome. Jobert syndrome is a polygenic disorder characterized by the presence of pathognomonic ‘molar tooth sign’ on brain magnetic resonance imaging, which is often accompanied by polydactyly, retinal and renal dysplasia. Hydrolethalus syndrome and acrocallosal syndrome are characterized by multiple digits, agenesis of the corpus callosum and facial abnormalities (Schinzel and Schmid, 1980; Salonen et al., 1981; Ibisler et al., 2015; Subramanian et al., 2019; Chen C. et al., 2021). Multiple KIF7 mutation sites have been identified in patient populations, including homozygous p.N1060S missense mutation (Ali et al., 2012), homozygous p.Glu779* meaningless mutation (Ibisler et al., 2015), and heterozygous mutations such as c.3365C > G and c.461G > a (Tunovic et al., 2015). These loci mutations will make patients present the typical features of the above three diseases, but mutations at different loci will also be accompanied by some atypical features, such as multiple epiphyseal dysplasia, growth retardation, etc., These results indicate that the mutations at different sites of KIF7 are related to the characteristics of the disease, and clinical judgment and targeted treatment can be carried out according to different mutation sites.

## 5 Summary and prospect

The KIFs, as a type of molecular motor proteins, are mainly involved in the transport of cellular materials along microtubules and biological processes such as spindle recombination and chromosome segregation during mitosis, playing a crucial role in maintaining the normal morphology and function of cells. With the in-depth research on KIFs, it has been discovered that multiple family members of KIFs play significant roles in neural development of the brain and have a considerable influence on the proliferation and differentiation of neural progenitor cells as well as the migration of neurons. At the same time, it should also be recognized that to fully elaborate the functions of kinesins, further efforts are still required. Many studies have identified genetic mutation sites and abnormal mRNA and protein expressions of KIFs members in various neurological disorders, suggesting that KIFs members may be candidate genes and markers for some neurological diseases. It has also been observed that multiple kinesin expression abnormalities or mutation sites can be detected in the same neurological disorder, indicating that the functional roles of KIFs members may be coordinated and complementary. Therefore, multi-gene joint research is necessary. It is hoped that in the future, in-depth studies on the functions and regulatory mechanisms of KIFs can be conducted through multi-gene edited cells or animal models (such as rodents and non-human primates), and gene and proteomic analyses can be carried out using knockout models to identify genes and proteins with abnormal expressions and the involved regulatory pathways. This will facilitate further exploration of the protein functions and molecular regulatory mechanisms of KIFs and the search for new therapeutic targets and regimens for related neurological diseases, as well as early identification indicators and treatment intervention plans. Hence, studies on the functional mechanisms of KIFs and their association with neurological disorders are of great significance for the clinical diagnosis and treatment of diseases.
